# Surface Charge Affects the Intracellular Fate and Clearance Dynamics of CdSe/ZnS Quantum Dots in Macrophages

**DOI:** 10.3390/nano15151189

**Published:** 2025-08-03

**Authors:** Yuan-Yuan Liu, Yong-Yue Sun, Yuan Guo, Lu-Lu Chen, Jun-Hao Guo, Haifang Wang

**Affiliations:** Institute of Nanochemistry and Nanobiology, Shanghai University, Shanghai 200444, China; yuan-yuanliu@shu.edu.cn (Y.-Y.L.); sunyongyue@shu.edu.cn (Y.-Y.S.); yguo1011@shu.edu.cn (Y.G.); clulu@shu.edu.cn (L.-L.C.); guojunhao810@shu.edu.cn (J.-H.G.)

**Keywords:** macrophages, quantum dots, surface charge, cellular uptake, exocytosis

## Abstract

The biological effects of nanoparticles are closely related to their intracellular content and location, both of which are influenced by various factors. This study investigates the effects of surface charge on the uptake, intracellular distribution, and exocytosis of CdSe/ZnS quantum dots (QDs) in Raw264.7 macrophages. Negatively charged 3-mercaptopropanoic acid functionalized QDs (QDs-MPA) show higher cellular uptake than positively charged 2-mercaptoethylamine functionalized QDs (QDs-MEA), and serum enhances the uptake of both types of QDs via protein corona-mediated receptor endocytosis. QDs-MEA primarily enter the cells through clathrin/caveolae-mediated pathways and predominantly accumulate in lysosomes, while QDs-MPA are mainly internalized through clathrin-mediated endocytosis and localize to both lysosomes and mitochondria. Exocytosis of QDs-MPA is faster and more efficient than that of QDs-MEA, though both exhibit limited excretion. In addition to endocytosis and exocytosis, cell division influences intracellular QD content over time. These results reveal the charge-dependent interactions between QDs and macrophages, providing a basis for designing biocompatible nanomaterials.

## 1. Introduction

Semiconductor quantum dots (QDs) have garnered significant attention in biomedical applications, including cellular imaging, drug delivery, and disease diagnosis, owing to their unique optical properties, such as size-tunable fluorescence emission, high quantum yield, and photostability [1,2,3,4,5]. Compared with other QDs such as CdSe, core–shell CdSe/ZnS QDs exhibit enhanced fluorescence stability, better biocompatibility, and easier surface modification, although they have a complex core–shell structure and potential long-term toxicity [6,7]. However, their clinical translation is hindered by macrophage-mediated immune clearance [8,9]. As key effector cells of innate immunity, Raw264.7 macrophages efficiently clear exogenous nanoparticles, making them the most commonly used in vitro model for preliminary investigation of QD–cell interactions, fates, and potential toxicities [10,11]. The response of Raw264.7 cells to QDs directly affects their circulation time, targeting accuracy, and long-term toxicity risks [10]. Therefore, understanding the dynamics of the interaction between QDs and Raw264.7 cells is crucial for optimizing their biocompatibility and enabling precision medicine applications.

The nanoparticle (NP)–macrophage interactions are influenced by many factors, including size, shape, and surface charge. For example, ultrasmall iron oxide NPs (USIONPs, 15.4 nm) exhibit more efficient endocytosis and exocytosis in Raw264.7 cells than their 7.8 nm and 30.7 nm counterparts. While 7.8 nm USIONPs localize predominantly near the nucleus, 15.4 nm USIONPs distribute broadly across organelles, including endosomes, the Golgi apparatus, lysosomes, mitochondria, and autophagosomes, highlighting size-dependent subcellular trafficking [11]. The shape of the NPs also modulates their uptake dynamics: long rod-shaped iron oxide nanoparticles (IONR(L), 140 nm × 6 nm) are internalized 1.36- and 1.17-fold more efficiently than short rods (IONR(S), 50 nm × 7 nm) or spherical NPs (20 nm), respectively. These disparities in shape correlate with distinct endocytic pathways; IONR(L) relies on clathrin-mediated, dynamin-dependent, and macropinocytic/phagocytic routes, whereas IONR(S) engages additional caveolae-mediated mechanisms [12]. Among these factors, surface charge plays a pivotal yet contentious role in NP uptake and intracellular fate. In most studies, positively charged NPs exhibit a stronger electrostatic attraction to the negatively charged macrophage membrane, leading to a higher cellular uptake [13,14,15]. For instance, cationic super-paramagnetic iron oxide NPs were internalized more efficiently by macrophages than their anionic or neutral counterparts [15]. Similarly, cholesterol-based acetylcarnitine-modified cationic liposomes showed enhanced uptake in Raw264.7 cells [13]. However, Zubareva et al. found that although negatively charged succinyl chitosan NPs had a lower binding affinity to the cell membrane than positively charged hexanoyl chitosan-based NPs, succinyl chitosan NPs were more efficiently internalized via endocytosis. Additionally, unlike cationic hexanoyl chitosan-based NPs, which were transported to lysosomes and mitochondria, anionic succinyl chitosan NPs exhibited more restricted lysosomal accumulation [16]. Cationic hyperbranched polymers not only entered the cytoplasm of Raw264.7 cells but also accumulated in the nucleus, whereas neutral and anionic hyperbranched polymers primarily localized to vesicular structures [17]. However, the impact of surface charge on the uptake of QDs has rarely been investigated.

In addition, current research is heavily biased towards uptake, and there is insufficient understanding of the exocytosis of NPs in Raw264.7 cells (such as residence time and efficiency). Studies have shown that due to their phagocytic properties, macrophages inherently have weak efflux capabilities [18]. The maximum efflux rate of alkyl-modified gold NPs is only 44% in Raw264.7 cells, which is significantly lower than in C166 cells (78%) and HeLa cells (88%) [18]. Moreover, exocytosis depends highly on NP properties, which are manifested in two aspects: exocytic pathways and exocytic efficiency. For example, USIONPs were exocytosed through the Golgi apparatus, recycling endosomes, and lysosomal pathways [11], while the lysosomal pathway was the only pathway for the exocytosis of single-walled carbon nanotubes [19]. Ho et al. reported that alkyl-modified gold NPs exhibited an efflux rate of only 10–44% in Raw264.7 cells, which is lower than in C166 (78%) and HeLa (88%) cells [18], but chitosan NPs had a higher efflux capacity in Raw264.7 cells than in HepG2 and A549 cells [20].

Charge is also one of the key factors influencing the exocytosis efficiency [21,22,23]. Positively charged gold NPs aggregate in cells, which hinders exocytosis (only 20–37% over 48 h), whereas charge regulation through polyethylene glycol functionalization significantly enhances exocytosis rates (71–83% over 48 h) [21]. Research on the impact of charge has so far concentrated almost entirely on traditional NPs (gold, polymers, etc.) [18,21,22,23], leaving a gap in our understanding of how the charge of QDs affects their exocytosis after uptake by Raw264.7 cells. Therefore, it is crucial to conduct a systematic study of the interactions (uptake, intracellular trafficking, and exocytosis) between QDs of different charges and Raw264.7 cells to elucidate charge-mediated uptake/efflux. This is helpful to optimize nanocarrier design and assess the long-term toxicity risks of QDs.

Therefore, leveraging the strong fluorescence of CdSe/ZnS QDs and considering the effect of cell division on NP uptake and exocytosis, this study systematically investigates the dynamic behavior of CdSe/ZnS QDs with different surface charges in Raw264.7 macrophages. The finding will help to elucidate the charge-dependent interactions between QDs and macrophages and provide valuable data for the design of low-toxicity, highly targeted nanoprobes.

## 2. Materials and Methods

### 2.1. Preparation of QDs-MEA and QDs-MPA

The core/shell CdSe/ZnS QDs were synthesized following the method reported by [24]. The QDs were functionalized with 2-mercaptoethylamine hydrochloride (MEA·HCl) and 3-mercaptopropanoic acid (MPA) following the literature [25,26], and are denoted as QDs-MEA and QDs-MPA, respectively. The morphology and size of the QDs were characterized by TEM (HT7700, Hitachi, Tokyo, Japan). The hydrodynamic size and ζ-potentials of the QDs were measured by a Nano ZS90 (Malvern, UK). The surface groups of the QDs were characterized by infrared spectroscopy (FT/IR-4100, Jasco, Tokyo,Japan). The absorption and fluorescence spectra were recorded using a UV spectrometer (U-3010, Hitachi, Tokyo, Japan) and a fluorescence spectrometer (F7000, Hitachi, Tokyo, Japan), respectively.

### 2.2. Cell Culture

The murine macrophage Raw264.7 cells, obtained from the Cell Bank of Type Culture Collection of Chinese Academy of Sciences (Shanghai, China), were cultured in high glucose (4.5 g/L glucose) Dulbecco’s modified Eagle medium (DMEM) supplemented with 20% (*v*/*v*) fetal bovine serum (FBS, Sigma-Aldrich, St. Louis, MO, USA) and 1% penicillin–streptomycin in a humidified incubator (37 °C, 5% CO_2_). Raw264.7 cells were seeded into 96-well plates (10 × 10^3^ cells/well, for the cell viability assay and fluorescence microscopic investigation), 12-well plates (3 × 10^5^ cells/well, for the flow cytometry analysis), or confocal laser scanning microscope (CLSM) dishes (d = 35 mm, 5 × 10^5^ cells/dish, for the CLSM investigation), and incubated overnight. The medium was then discarded, and the cells were exposed to the QDs suspended in the culture medium for a period of time. This was followed by different assays.

### 2.3. Cell Viability Assay

Cell viability was measured using the CCK-8 kit (CCK-8; Beyotime Biotechnology Co., Ltd., Shanghai, China). Raw264.7 cells were exposed to the culture medium (with/without 20% FBS) containing the QDs at different concentrations for 24 h. Subsequently, the medium was abandoned and the CCK-8 solution (10%, 100 μL) was added to each well. After 1 h incubation, the optical density of each well at 450 nm was recorded on a microplate reader (Varioskan Flash, Thermo Fisher Scientific, Waltham, MA, USA).

### 2.4. Cellular Uptake Measured by Flow Cytometry

Cells were cultured in the medium (with/without 20% FBS) containing 25 μg/mL QDs for a predetermined period of time. Subsequently, the medium was aspirated and the cells were washed three times with cold D-Hank’s buffer, detached with trypsin, counted, and then suspended in 400 μL of the culture medium containing 20% FBS. Next, the cells were collected and suspended in 200 μL of D-Hank’s buffer for flow cytometry analysis (excitation at 488 nm). The uptake of QDs per cell is represented by the mean fluorescence intensity (FI) based on 20,000 cells. Total cell uptake is represented by the total FI of cells, which is equal to the mean FI multiplied by the number of cells counted after exposure.

To reveal the endocytic pathways of the QDs, the cellular uptake was measured at a low temperature or after the cells had been pretreated with endocytic inhibitors. The cells were first incubated at 4 °C or 37 °C for 30 min, then incubated in the medium (with/without 20% FBS) containing 25 μg/mL QDs for 2 h at the same temperature. Separately, the cells were pre-incubated with different endocytic inhibitors for 30 min, and then incubated for 2 h in the medium (with/without 20% FBS) containing the same inhibitor at the same concentration plus 25 μg/mL QDs. Subsequently, the cells were treated and analyzed by flow cytometry as described above. Inhibitors included chlorpromazine hydrochloride (CPZ, 7 μg/mL) (Innochem Co., Ltd., Beijing, China), Amantadine (100 μM) (Shanghai Aladdin Biochemical Technology Co., Ltd., Shanghai, China), and Nocodazole (15 μM) (Shanghai Aladdin Biochemical Technology Co., Ltd., Shanghai, China) for clathrin-mediated endocytosis, Filipin III (2.5 μg/mL) (APExBIO Technology LLC, Houston, TX, USA), methyl-β-cyclodextrin (MβCD, 2 mM) (Sigma-Aldrich, Louis, MO, USA), and Genistein (50 μg/mL) (Beijing Innochem Technology Co., Ltd., Beijing, China) for caveolae-mediated endocytosis, and Amiloride (50 μM) (Innochem Co., Ltd., Beijing, China) for macropinocytosis [27,28,29]. Cell viability experiments and cell morphology investigation under a microscope indicate that the inhibitors were non-toxic to Raw264.7 cells at the tested concentrations.

### 2.5. Cellular Uptake and Subcellular Localization Investigated by Fluorescence Microscope

Cells were cultured for 2 h in the medium (with 20% FBS) containing the QDs at different concentrations. Then, the medium was discarded, and cells were washed three times with cold D-Hank’s buffer. After that, the cells were cultured in fresh medium (with 20% FBS) and observed using a DMI3000 fluorescence microscope (Leica, Wetzlar, Germany).

Cells seeded in CLSM dishes were cultured in the medium (with 20% FBS) containing 25 µg/mL QDs for 2 h, 4 h, and 6 h. After discarding the medium, cells were incubated in 10 µg/mL Hoechst 33342 (excitation at 350 nm/emission at 460 nm) for 30 min at 37 °C. Then, the cells were washed with cold D-Hank’s buffer and maintained in 1 mL of fresh medium (with 20% FBS) for imaging using an Olympus FM 1000 (Olympus, Tokyo, Japan).

To determine the location of the QDs in cells, Raw264.7 cells were incubated in the medium (20% FBS) containing 25 μg/mL QDs for 2 h, 4 h, and 6 h. Next, the medium was discarded and the cells were exposed to the D-Hank’s buffer containing either 100 nM of LysoTracker-Red DND-26 (excitation at 577 nm/emission at 590 nm) or 100 nM of MitoTracker-Red FM (excitation at 581 nm/emission at 644 nm). Subsequently, the cells were washed with D-Hank’s buffer and maintained in 1 mL fresh medium (with 20% FBS) for imaging using the Olympus FM 1000.

### 2.6. Exocytosis Measured by Flow Cytometry

Cells were cultured in the medium (with 20% FBS) containing 25 μg/mL QDs for 12 h. After this time, the cells were washed three times with cold D-Hanks and cultured in fresh culture medium (with 20% FBS) in the incubator. At predetermined time points, the cells were harvested, counted, and suspended in 200 μL of D-Hank’s buffer for flow cytometric analysis (excitation at 488 nm; 20,000 cells were tested). Exocytosis of QDs per cell is expressed as the percentage of the mean FI of cells at a certain time point relative to the mean FI of cells immediately prior to exocytosis, and total exocytosis of QDs is expressed as the percentage of total FI of cells at a certain time point relative to the total FI of cells immediately prior to exocytosis, in which the total FI of cells is equal to the mean FI multiplied by the number of cells counted at the corresponding time point.

### 2.7. Statistical Analysis

All means were calculated from three parallel experiments, and the data are expressed as the mean ± standard deviation. One-way analysis of variance (ANOVA) followed by a post hoc test was used to test the statistical significance of differences between groups, and the assumptions of normality and homogeneity were tested (Prism9.5.0, GraphPad Software, San Diego, CA, USA). A value of *p* < 0.05 is considered statistically significant.

## 3. Results

### 3.1. Characterization of the QDs

Two types of CdSe/ZnS QDs, QDs-MEA (MEA = 2-mercaptoethylamine) and QDs-MPA (MPA = 3-mercaptopropanoic acid), were synthesized to study the effect of surface charge on the uptake and exocytosis of QDs. The functionalization of the QDs with MEA and MPA was confirmed by their infrared spectra (Figure S1). The N–H stretching of secondary N-substituted amide at around 3450–3225 cm^−1^ with a peak at 3321 cm^−1^, and the C–H asymmetric stretching at 2930 cm^−1^ suggested that MEA had been successfully functionalized on the surface of the QDs. The peaks at 1693 cm^−1^ and 1407 cm^−1^ correspond to the asymmetric and symmetric stretching vibrations of the carboxyl group, respectively, indicating that MPA was successfully grafted onto the surface of the QDs.

**Figure 1:**
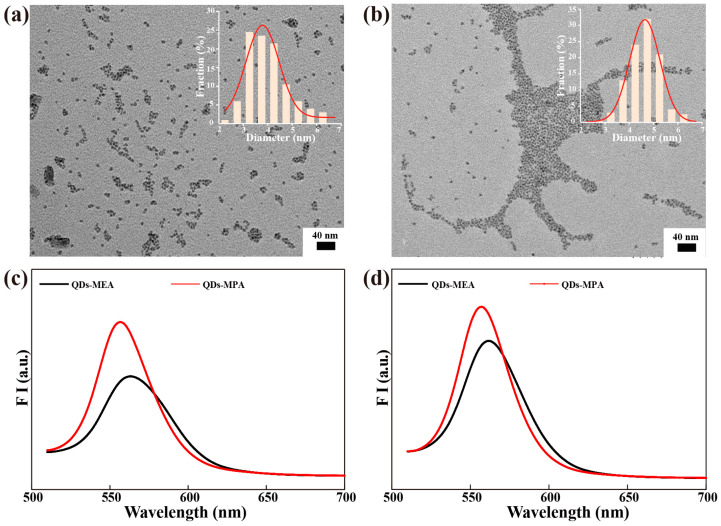
Properties of the QDs. (**a**,**b**) Representative TEM image and size distribution: (**a**) QDs-MEA, (**b**) QDs-MPA. (**c**,**d**) Fluorescence spectra (Excitation at 488 nm) of the QDs (25 µg/mL) in the medium without serum (**c**) and with serum (**d**).

The TEM images show that QDs-MEA and QDs-MPA are non-standard spherical shapes (Figure 1a,b), with an average particle size of 4.0 nm for QDs-MEA (Figure 1a) and 4.6 nm for QDs-MPA (Figure 1b). In aqueous solutions, both QDs agglomerated and exhibited different characteristics (Table 1). QDs-MEA agglomerated in water, as demonstrated by a hydrodynamic size of approximately 38 nm, which increased slightly over time. The size of QDs-MEA increased to approximately 47 nm and 50 nm in the medium with and without serum, respectively, but the particle size remained unchanged within 24 h, suggesting stability of QDs-MEA in the medium. The ζ-potential of QDs-MEA was positive and similar in water and medium. However, in the medium with serum, the ζ-potential became negative due to the formation of a protein corona on the surface of QDs-MEA. As shown in Table 1, QDs-MPA had a slightly larger size and poorer stability than QDs-MEA, probably due to the different protein corona formed on QDs-MPA. In the medium with serum, the hydrodynamic size of QDs-MPA increased from 51 nm to about 72 nm over 24 h. The ζ-potential of QDs-MPA was negative in all three dispersions, and the ζ-potential was similar to that of QDs-MEA in the medium with serum.

The fluorescence spectra of QDs in different aqueous solutions are shown in Figure 1c,d and Figure S2. In water, the emission peak of both types of QDs was at about 553 nm, and the FI of QDs-MEA was 2.6 times that of QDs-MPA at equal concentration. However, the FI of QDs-MPA was 1.5 and 1.2 times that of QDs-MEA in the medium without serum (Figure 1c) and with serum (Figure 1d), respectively. The difference in FI enhancement between the two types of QDs in medium with serum likely reflects the different protein corona formed on the QDs, which alter the QDs’ environment and dispersion differently (Table 1). In addition, QDs-MEA exhibited a shoulder at longer wavelengths in the medium without serum, likely reflecting heterogeneous aggregation states, as evidenced by their wider hydrodynamic size distribution (Table 1). Both types of QDs showed narrower emission peaks in medium with serum (Figure 1d), indicating improved colloidal homogeneity through the formation of protein corona. Overall, these spectral variations primarily arise from the modulation of the dispersion state of the QDs by the environment [30].

### 3.2. Cell Viability

The effect of the QDs on the viability of Raw264.7 cells is shown in Figure S3. After cells were exposed to the QDs (0 to 50 μg/mL) in the medium with serum for 24 h, the viability remained above 95% (Figure S3a). In serum-free medium, the viability remained above 85% after 12 h of exposure to the QDs (Figure S3b). These results suggested that QDs-MEA and QDs-MPA do not significantly affect cell viability under experimental conditions of this study; therefore, the subsequent cell experiments were performed at safe doses.

### 3.3. Cellular Uptake of the QDs

**Figure 2:**
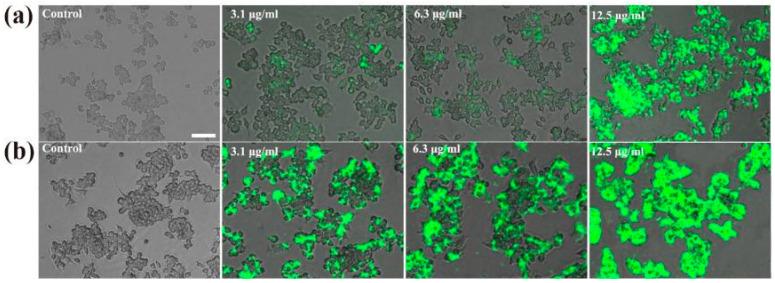
Fluorescence microscopic images of Raw264.7 cells after incubation with QDs for 2 h in the medium with serum (green: QDs). (**a**) QDs-MEA. (**b**) QDs-MPA. Scale bar: 40 μm (for all panels).

The cellular uptake of QDs-MEA and QDs-MPA was first investigated using a fluorescence microscope. As shown in Figure 2, with the increase in QD concentration, the uptake of both types of QDs by Raw264.7 cells increased, and QDs-MPA showed significantly higher uptake than QDs-MEA at all test concentrations.

**Figure 3:**
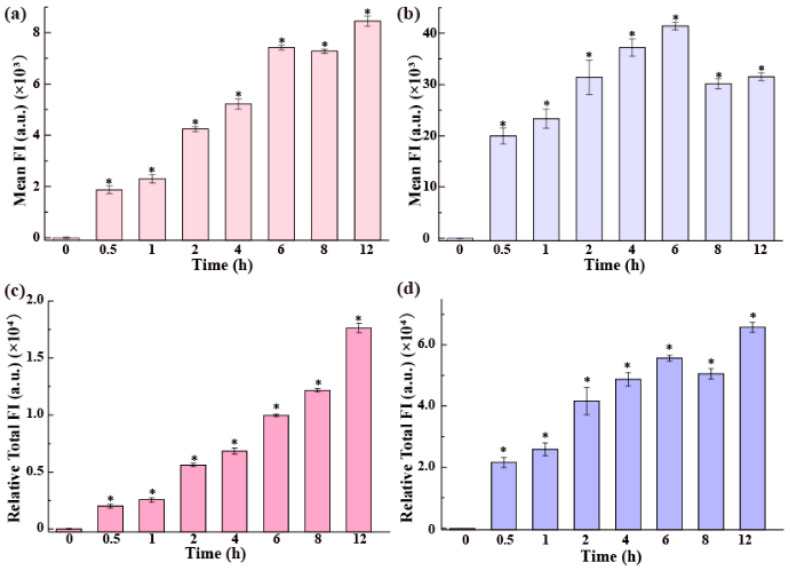
Uptake of the QDs by Raw264.7 cells measured by flow cytometry. Cells were exposed to 25 μg/mL QDs for different times in the medium with serum. (**a**,**b**) Uptake is represented by the mean FI per cell: (**a**) QDs-MEA, (**b**) QDs-MPA. (**c**,**d**) Uptake is represented by the relative total FI in all cells: (**c**) QDs-MEA, (**d**) QDs-MPA. * *p* < 0.05 vs. the control (n = 3).

Besides the dose of QDs, culture time was also a critical factor influencing cellular uptake. We measured the cellular uptake of the QDs in the medium with serum using flow cytometry by detecting the FI of 20,000 cells and calculating the mean FI per cell. The results are shown in Figure 3a,b. In the medium containing serum, the uptake of QDs-MEA by Raw264.7 cells increased over time, slowing down after 6 h (Figure 3a). A different uptake profile was observed for QDs-MPA, which showed a peak at 6 h (Figure 3b). After correcting for the FI ratio of QDs-MEA to QDs-MPA (1.2 at equal mass concentration), the mass of QDs-MPA per cell was found to be 4.6 times that of QDs-MEA at 6 h, demonstrating a significantly higher cellular uptake of QDs-MPA than that of QDs-MEA, which is consistent with the results of the fluorescence microscopic investigation (Figure 2).

As the cell proliferation influences the intracellular content of the QDs [31,32,33,34], at different time points, we counted the cells before the flow cytometry measurement. As shown in Figure S4a, the number of cells increased over time, especially after 6 h. We therefore calculated the relative total QD uptake by all cells using cell number correction, i.e., setting the number of cells at 0 h as 1. As shown in Figure 3c, the uptake of QDs-MEA by Raw264.7 cells increased continuously without saturation or peak within 12 h. As for QDs-MPA, in contrast to the profile of uptake per cell (Figure 3b), the total uptake by all cells also increased continuously within 12 h (Figure 3d). These results suggest that cell division should be considered in the study of cell uptake, especially when incubation time exceeds 6 h, which was also suggested by other researchers [35]. At each time point, Raw264.7 cells internalized more QDs-MPA than QDs-MEA, indicating that they internalized QDs-MPA more rapidly and efficiently than QDs-MEA. Based on the FIs of QDs-MEA and QDs-MPA after 12 h, the total intracellular QDs-MPA was 3.1 times that of QDs-MEA.

**Figure 4:**
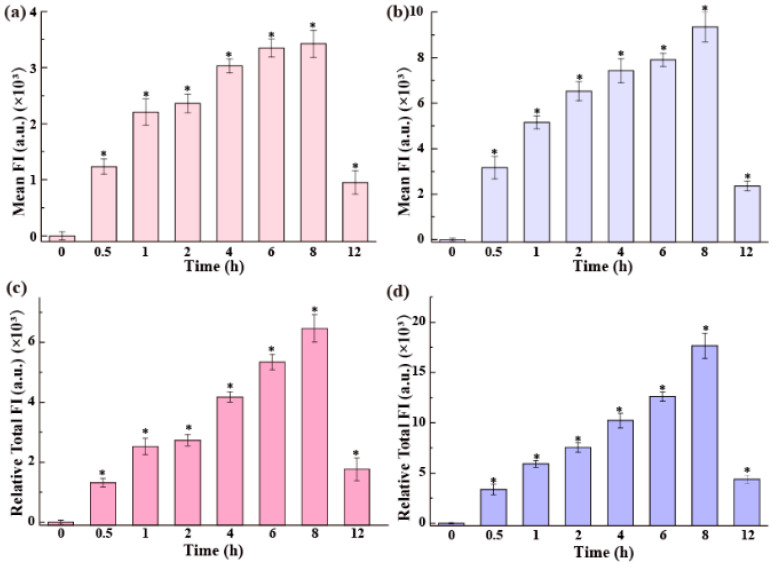
Uptake of the QDs by Raw264.7 cells measured by flow cytometry. Cells were exposed to 25 μg/mL QDs for different times in the medium without serum. (**a**,**b**) Uptake is represented by the mean FI per cell: (**a**) QDs-MEA, (**b**) QDs-MPA. (**c**,**d**) Uptake is represented by the relative total FI in all cells: (**c**) QDs-MEA, (**d**) QDs-MPA. * *p* < 0.05 vs. the control (n = 3).

In the serum-free medium, as shown in Figure 4a,b, the cellular uptake of the QDs by Raw264.7 cells increased over time and peaked at 8 h. Although the uptake of QDs-MPA was still higher than that of QDs-MEA, the uptake of both QDs was inhibited in the medium without serum. The inhibition of QDs-MPA uptake was stronger than that of QDs-MEA. The amount of QDs-MPA per cell was 1.8 times that of QDs-MEA at 8 h in the medium without serum, but 4.7 times at 6 h in the medium with serum (Figure 3a,b). At 12 h, the intracellular QD content decreased dramatically. Similarly, we corrected the uptake values with cell numbers (Figure S4b) and found that the total amount of QDs-MEA and QDs-MPA internalized by all cells still peaked at 8 h, followed by a sharp decrease at 12 h. The sharp decrease in the QD content in the Raw264.7 cells may be due to increased exocytosis of QDs over time, as well as a drop in uptake capacity in the medium without serum, particularly over a longer period. Further investigation is required.

### 3.4. Mechanisms of Cellular Uptake

To better understand the uptake process of the two types of QDs by Raw264.7 cells, the associated mechanistic issues were explored by using low-temperature treatment and endocytic inhibitors.

**Figure 5:**
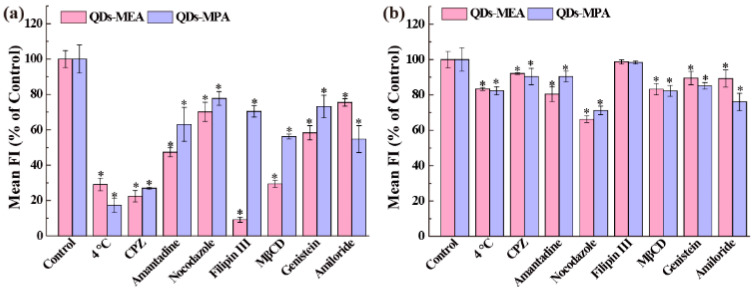
Mechanisms involved in the uptake of the two types of QDs by Raw264.7 cells in the medium with serum (**a**) or without serum (**b**). * *p* < 0.05 vs. the control (n = 3).

In the culture medium with serum, the cellular uptake of the QDs by Raw264.7 cells was primarily energy-dependent, as the cellular uptake at 4 °C decreased dramatically to approximately 20% of the control (Figure 5a). Furthermore, after treatment with clathrin-mediated endocytic inhibitors (CPZ, Amantadine, and Nocodazole), caveolae-mediated endocytic inhibitors (Filipin III, MβCD, and Genistein), or a macropinocytosis inhibitor (Amiloride), the cells always lowered their uptake capability for QDs (Figure 5a). That is, clathrin-mediated endocytosis, caveolae-mediated endocytosis, and macropinocytosis all played a role in the uptake of two types of QDs by Raw264.7 cells. It was observed that CPZ, Filipin III, and MβCD produced the most significant inhibition of QDs-MEA uptake, whereas CPZ produced the most significant inhibition of QDs-MPA uptake. The results indicated that the QDs entered the cells via multiple pathways. For QDs-MEA, the pathways were as follows: caveolae-mediated endocytosis, clathrin-mediated endocytosis, and macropinocytosis. In contrast, the primary endocytic pathway for QDs-MPA was clathrin-mediated endocytosis, followed by caveolae-mediated endocytosis and macropinocytosis. The latter two pathways exhibited comparable contributions.

In the culture medium without serum, the cellular uptake of the two types of QDs by Raw264.7 cells was primarily non-energy dependent. QDs-MEA primarily entered Raw264.7 cells through clathrin-mediated endocytosis, followed by caveolae-mediated endocytosis and macropinocytosis, with the latter two pathways exhibiting comparable contributions. The dominant endocytic pathway for QDs-MPA was clathrin-mediated endocytosis, followed by macropinocytosis, and finally caveolae-mediated endocytosis (Figure 5b).

### 3.5. Intracellular Localization of the QDs

**Figure 6:**
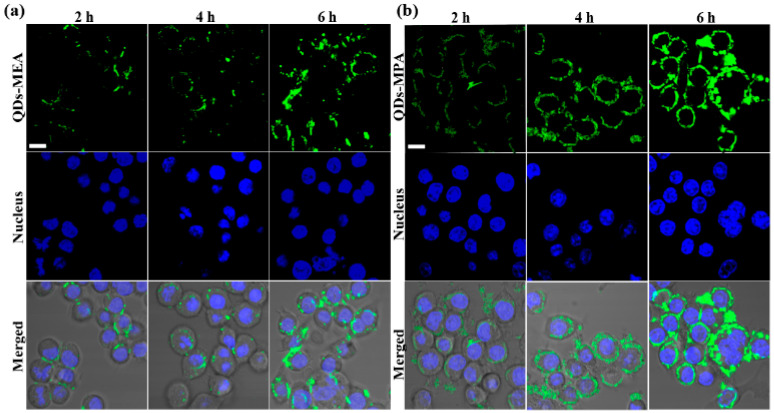
CLSM images of Raw264.7 cells after exposure to QDs (25 μg/mL) in the medium with serum for 2 h, 4 h, and 6 h. (**a**) QDs-MEA. (**b**) QDs-MPA. Nuclei were stained with Hoechst 33342 (blue). The scale represents 40 μm (for all panels).

**Figure 7:**
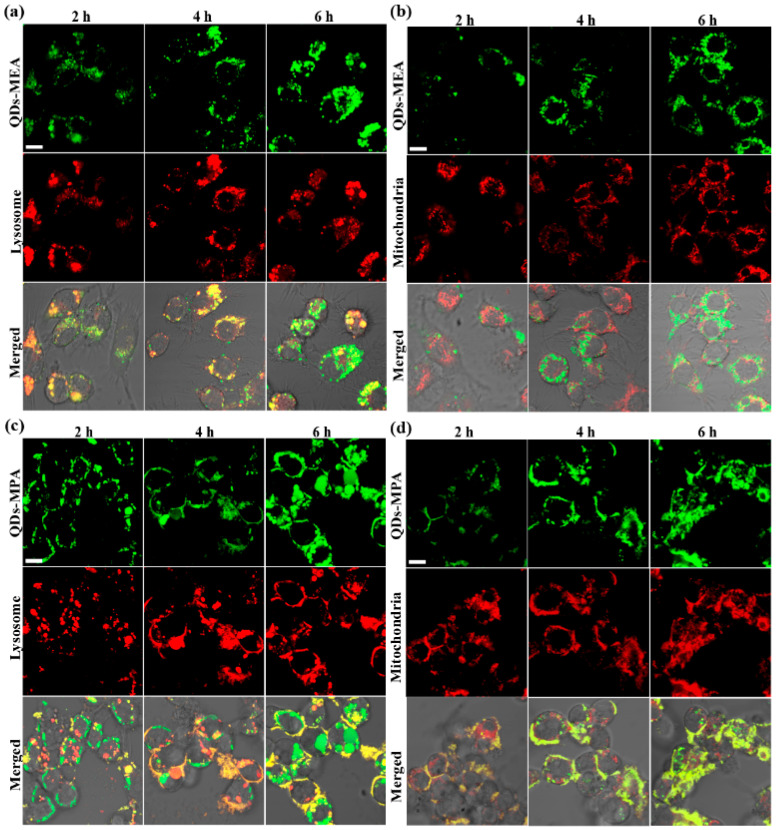
CLSM images of Raw264.7 cells after exposure to QDs (25 μg/mL) in the medium with serum for 2 h, 4 h, and 6 h. (**a**,**b**) cells were exposed to QDs-MEA (green). (**c**,**d**) cells were exposed to QDs-MPA (green). Lysosomes (**a**,**c**) and mitochondria (**b**,**d**) are labelled with red. The scale represents 40 μm (for all panels).

Figure 6 and Figure 7 show the locations of the QDs in Raw264.7 cells after incubation in medium with serum for 2 h to 6 h. As in Figure 2, more QDs-MPA were found in Raw264.7 cells than QDs-MEA, and the QDs in the cells increased over time (Figure 6). In addition, after entering the cells, both types of QDs moved from the cell periphery to the perinuclear region, but did not enter the nucleus (Figure 6).

QDs-MEA and QDs-MPA were found in different organelles within the cells. After 2 h, significant overlap was observed between the signals of QDs-MEA and lysosomes (Figure 7a). After 4 h, more QDs-MEA signals (green) overlapped with lysosome signals (red), indicating that the endosome-lysosome was the main organelle involved in transporting QDs-MEA. However, more QDs-MEA left the lysosomes after 6 h. Interestingly, as shown in Figure 7b, most QDs-MEA were not co-localized with the mitochondria within the 6 h incubation period, indicating that the mitochondria were not the destination of QDs-MEA. QDs-MPA behaved very differently from QDs-MEA. At 2 h, there was significant overlap between the signals of QDs-MPA and mitochondria, and over time, an increasing number of QDs-MPA reached the mitochondria (Figure 7d). However, only a few of the QDs-MPA were co-localized with lysosomes at 2 h, and more QDs-MPA entered lysosomes at 4 h, although some left lysosomes at 6 h (Figure 7c). Therefore, both lysosomes and mitochondria were destinations of QDs-MPA.

### 3.6. Exocytosis of the QDs

**Figure 8:**
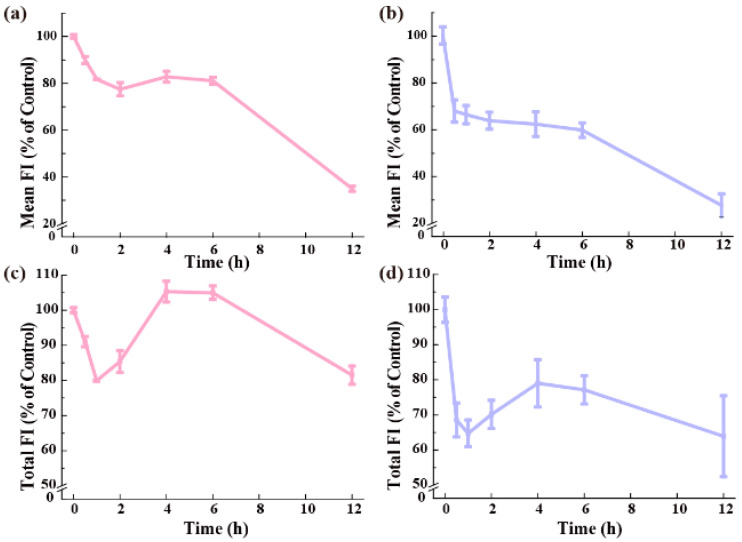
Exocytosis of the QDs from Raw264.7 cells after cells had been preincubated with 25 μg/mL QDs in the medium with serum for 12 h (n = 3). (**a**,**b**) Residual QDs in cells, expressed as the mean FI (% of control): (**a**) QDs-MEA; (**b**) QDs-MPA. (**c**,**d**) Exocytosis of QDs, expressed as the total FI (% of control): (**c**) QDs-MEA; (**d**) QDs-MPA.

The exocytosis of the QDs in cells was investigated in the fresh medium once the maximum intracellular contents of QDs had been reached in the medium with serum after 12 h of preincubation (Figure 3a). Figure 8a shows the percentage of remaining QDs-MEA in the cells during the exocytosis phase. Intracellular QDs-MEA levels decreased significantly within the first 2 h, with an excretion rate approaching 25%. Then, the exocytosis entered a dormant phase from 2 h to 6 h. After 6 h, the decline continued, with around 70% of the intracellular QDs-MEA expelled by 12 h. Given that cell division also results in a decrease in intracellular QDs-MEA, we counted the number of cells at various time points during the exocytosis phase (Figure S5) and calculated the total amount of intracellular QDs-MEA in the cells (Figure 8c), which is equal to the difference between 100% and the exocytosis percentage. A very different profile was observed, highlighting the significance of cell division. Exocytosis was significant in the first hour; about 20% of the total intracellular QDs-MEA was expelled. After that, the total amount of QDs-MEA in the cells increased significantly, reaching the initial level after 4–6 h. After 6 h, the exocytosis became significant, with approximately 20% of the total intracellular QDs-MEA being expelled after 12 h.

Intracellular QDs-MPA behaved similarly to QDs-MEA during the exocytosis phase (Figure 8b,d). The main difference was that the exocytosis of QDs-MPA was much faster and easier than that of QDs-MEA. Intracellular QDs-MPA decreased to 65% of the initial amount after 30 min of exocytosis, dropping to 25% after 12 h (Figure 8b). After correcting for cell number (Figure S5), it can be seen that significant exocytosis (35% of the initial amount) occurred within the first hour. Thereafter, significant re-uptake occurred, most probably by cells that had just undergone division, resulting in the total intracellular content of QDs-MPA reaching a peak at 4 h (approximately 77% of the initial amount). Similar to QDs-MEA, the exocytosis at 12 h was comparable to that at 1 h.

## 4. Discussion

The disparity in NP uptake between macrophages and non-phagocytic cells primarily stems from their functional specificity. Studies have demonstrated that macrophages exhibit significantly stronger NP internalization than non-phagocytic cells (e.g., HeLa cells) due to their professional phagocytic capability [18,20,36]. Our results align with this observation, i.e., Raw264.7 cells showed markedly higher uptake of QDs-MEA than HeLa cells, in both culture medium with and without serum [32]. Notably, this difference in uptake efficiency was not limited to QDs-MEA, since Raw264.7 cells internalized QDs-MPA more efficiently than QDs-MEA (Figure 3 and Figure 4), suggesting that macrophages possess a high capacity for engulfing NPs with different surface modifications. In fact, surface charge is a critical factor in NP uptake by macrophages. Previous studies have demonstrated that macrophages preferentially internalize negatively charged NPs, including CdSe/CdS/ZnS QDs [37,38]. Consistent with this, we observed a higher uptake of negatively charged QDs-MPA than positively charged QDs-MEA (Figure 3 and Figure 4). Lunov et al. proposed that negatively charged particles are opsonized by IgG and recognized by CD64 receptors, thereby triggering efficient phagocytosis, whereas positively charged particles are internalized via non-Fc receptor-dependent pathways (e.g., macropinocytosis or caveolin-mediated endocytosis), which are less efficient [37]. In serum-free conditions, the uptake of both types of QDs was reduced (Figure 3 and Figure 4). This is likely due to the absence of serum opsonization, which reduces phagocytic efficiency.

Indeed, serum exerts opposing effects on NP uptake in macrophages and non-phagocytic cells. We found that serum significantly increased the uptake of both QDs in Raw264.7 cells (Figure 3 and Figure 4), which contrasts with our previous observations in HeLa cells, where serum reduced QDs-MEA uptake [32]. This discrepancy may be attributed to the different roles played by the protein corona formed on NPs when they interact with different cell types. In non-phagocytic cells, the protein corona likely shields the positive surface charge of QDs-MEA, reducing electrostatic interactions with the cell membrane and consequently decreasing uptake [32]. By contrast, specific serum proteins (e.g., the Fc region of immunoglobulins or complement proteins) may bind to cell surface receptors (Fc receptors or complement receptors) in macrophages, thereby facilitating receptor-mediated endocytosis [37]. A similar phenomenon was observed with citraconic anhydride-modified exosomes, which bind to complement protein C3b and trigger efficient phagocytosis via complement receptors (CR1, CR3), enhancing the uptake of citraconic anhydride-modified exosomes by Raw264.7 cells [39].

Internalization pathway analysis revealed that QDs-MEA primarily enter cells via caveolin- and clathrin-mediated pathways, whereas QDs-MPA uptake involves all clathrin- and caveolin-mediated endocytosis, as well as macropinocytosis, in serum-containing medium (Figure 5a). This is consistent with previous studies [40]. Raw264.7 cells can internalize chitosan NPs (CsNPs, ~250 nm) via multiple pathways, including clathrin-mediated endocytosis, macropinocytosis, in addition to phagocytosis. For the uptake of single-walled carbon nanotubes (SWCNTs) by Raw264.7 cells, macropinocytosis plays the primary role, followed by caveolae-mediated and clathrin-dependent pathways, with the clathrin-mediated pathway being particularly important for shorter SWCNTs [19]. Concurrently, culture medium significantly affects QD uptake (Figure 5). In serum-containing medium, the uptake of the QDs by Raw264.7 cells is primarily an energy-dependent process, as demonstrated by a dramatic reduction to ~20% of the control level at 4 °C (Figure 5a). Serum proteins that are adsorbed onto the QDs facilitate endocytosis via multiple pathways, including clathrin-mediated, caveolae-mediated, and macropinocytic mechanisms. Specifically, QD-MEA uptake mainly relies on both clathrin- and caveolae-mediated pathways (with the latter making the greatest contribution), whereas QD-MPA internalization is predominantly driven by clathrin-mediated endocytosis. Conversely, in serum-free medium, the uptake mechanism becomes energy-independent with minimal temperature sensitivity at 4 °C. The absence of serum proteins might trigger the activation of alternative pathways (e.g., non-energy-dependent routes) as a compensatory response, resulting in opposing trends in the efficacy of the clathrin pathway compared to serum-containing conditions. Consequently, the intracellular localization of the QDs differed significantly: QDs-MEA predominantly accumulated in lysosomes (Figure 7b), whereas QDs-MPA localized to both lysosomes and mitochondria (Figure 7c,d). QDs-MEA that were internalized via clathrin-mediated endocytosis (Figure 5a) would be transported through early endosomes to lysosomes (Figure 7a), while QDs-MEA that were internalized via caveolin-mediated endocytosis would be delivered to other organelles, such as the endoplasmic reticulum [32,33,41]. As for QDs-MPA, clathrin-mediated endocytosis led to their accumulation in lysosomes. MPA on QDs may facilitate escape to the cytoplasm and interaction with mitochondria [42]. However, our results contradict those of some researchers, who found that smaller, positively charged NPs more readily target mitochondria [38,43]. This suggests that factors beyond surface charge (e.g., NP size and surface chemistry) may influence the subcellular distribution of NPs.

The uptake of the QDs by Raw264.7 cells exhibited a dose-dependent increase within the tested range (Figure 2), which is consistent with the observations using other NPs [19,20,44]. Time-course experiments showed that, in serum-containing media, QDs-MEA uptake increased continuously over 12 h, whereas QDs-MPA uptake peaked after 6 h (Figure 3a). In serum-free media, the uptake of both types of QDs peaked after 8 h (Figure 3b and Figure 4a,b). These differences may reflect the dynamic equilibrium between cellular division, uptake, and exocytosis.

Both types of QDs were inefficiently excreted from Raw264.7 cells. The initial rapid excretion phase observed within the first hour (Figure 8) primarily reflects the fast exocytosis of intracellular QDs near the cell membrane, as well as the QDs embedded in the cell membrane. This exocytic profile is consistent with established nanoparticle trafficking mechanisms in phagocytic cells [11]. QDs-MPA were expelled at a higher rate and to a greater extent than QDs-MEA (Figure 8). This is consistent with the findings of Oh et al., who reported that cationic gold NPs were retained longer and excreted more slowly than their anionic or zwitterionic counterparts [21], although some studies have reported that cationic NPs are much more easily released from MCF-7/ADR cells than anionic NPs [22,45]. While studies on the exocytosis process of Cd-containing QDs remain limited, it has been found that their exocytosis is affected by many factors, including size, surface charge, dose, and cell type and species [46]. For example, we found that QDs-MEA was more easily excreted from HeLa cells than Raw264.7 cells [32]

Interestingly, an initial rapid excretion phase was followed by a transient increase in intracellular QD content (particularly for QDs-MEA). This phenomenon may arise from a combination of reversed exocytosis and endocytosis, whereby cells re-internalize extracellular QDs after initial expulsion [47,48,49]. NPs can be exocytosed freely or enclosed inside extracellular vesicles for re-entry [48]. Ultimately, it was demonstrated that QDs were expelled from cells (Figure 8c,d). These dynamics highlight the importance of considering both excretion rates and cell proliferation when evaluating NP efflux behavior.

## 5. Conclusions

In this study, we studied the uptake, subcellular distribution, and exocytosis of QDs in Raw264.7 cells and examined the effect of QD surface charge on these processes. We found that Raw264.7 cells easily engulfed QDs, and that this increased with dose and culture time. The negatively charged QDs-MPA showed a higher potential to be engulfed by Raw264.7 cells than the positively charged QDs-MEA, particularly in serum-containing medium, where protein corona formation promoted receptor-mediated endocytosis. The QDs entered Raw264.7 cells primarily via an energy-dependent process: QDs-MEA could be internalized via a combination of clathrin/caveolae-mediated endocytosis and preferentially entered lysosomes, while QDs-MPA entered via clathrin-mediated endocytosis and accumulated in lysosomes and mitochondria, reflecting charge-driven subcellular sorting. In the absence of serum, both types of QDs entered cells primarily in an energy-independent manner. Both types of QDs exhibited poor excretion, but QDs-MPA were cleared faster than QDs-MEA. These results highlight the importance of surface charge as a critical design parameter for controlling nanoparticle–macrophage interactions, providing a foundation for developing biocompatible QDs.

## Figures and Tables

**Table 1:** Hydrodynamic sizes and ζ-potentials of the QDs.

Dispersing Agent	Time (h)	Size (nm)		ζ-Potential (mV)	
		QDs-MEA	QDs-MPA	QDs-MEA	QDs-MPA
Water	0	38.2 ± 7.6	48.2 ± 3.5	22.3 ± 3.4	−20.7 ± 1.0
	12	39.7 ± 9.2	51.2 ± 6.7	26.7 ± 3.0	−24.5 ± 4.8
	24	48.9 ± 6.4	53.4 ± 9.8	27.8 ± 4.9	−22.5 ± 5.6
DMEM	0	46.7 ± 4.3	49.8 ± 7.6	23.4 ± 4.3	−23.4 ± 7.8
	12	43.4 ± 3.4	52.5 ± 6.3	25.6 ± 3.8	−27.4 ± 4.5
	24	47.8 ± 4.5	54.2 ± 5.1	27.5 ± 3.4	−23.7 ± 4.3
DMEM (20% FBS)	0	49.5 ± 3.5	51.2 ± 3.8	−12.4 ± 3.2	−7.7 ± 3.4
	12	49.7 ± 4.2	61.7 ± 7.8	−11.7 ± 2.8	−12.8 ± 7.2
	24	53.2 ± 4.3	72.4 ± 5.6	−8.8 ± 0.5	−11.8 ± 5.2

## Data Availability

Data are available from the authors upon reasonable request.

## Supplementary Materials

The following supporting information can be downloaded at: https://www.mdpi.com/article/10.3390/nano15151189/s1, Figure S1: Infrared spectra of the QDs; Figure S2: Fluorescence spectra of the QDs; Figure S3: Viability of Raw264.7 cells after exposure to the QDs; Figure S4: Cell number of Raw264.7 cells after exposed to the QDs; Figure S5: Cell number of Raw264.7 cells during the exocytosis phase.

## Abbreviations

CLSM: confocal laser scanning microscope; CPZ: chlorpromazine hydrochloride; DMEM: Dulbecco’s modified Eagle medium; FBS: fetal bovine serum; FI: fluorescence intensity; IONR: rod-shaped iron oxide nanoparticles; MEA·HCl: 2-mercaptoethylamine hydrochloride; MPA: 3-mercaptopropanoic acid; MβCD: methyl-β-cyclodextrin; NPs: nanoparticles; QDs: quantum dots; QDs-MEA: 2-mercaptoethylamine functionalized QDs; QDs-MPA: 3-mercaptopropanoic acid functionalized QDs; SWCNTs: single-walled carbon nanotubes; USIONPs: ultrasmall iron oxide nanoparticles.
