# Supplementary material for: Surface Charge Affects the Intracellular Fate and Clearance Dynamics of CdSe/ZnS Quantum Dots in Macrophages
Source: Nanomaterials (Basel). 2025 Aug 3;15(15):1189. doi: 10.3390/nano15151189 (PMC12348891; doi:10.3390/nano15151189)
Supplement: Supplementary file 1 [file nanomaterials-15-01189-s001.zip › nanomaterials-3753271-supplementary.pdf]

nanomaterials

Supplementary Materials

Surface Charge Affects the Intracellular Fate and Clearance Dynamics of CdSe/ZnS Quantum Dots in Macrophages

Yuan-Yuan Liu[1,†], Yong-Yue Sun[1,†], Yuan Guo[1], Lu-Lu Chen[1], Jun-Hao Guo[1], and Haifang Wang[1,*]

[1] Institute of Nanochemistry and Nanobiology, Shanghai University, Shanghai 200444, China
* Correspondence: hwang@shu.edu.cn (H. Wang)
† These authors contributed equally to this work.

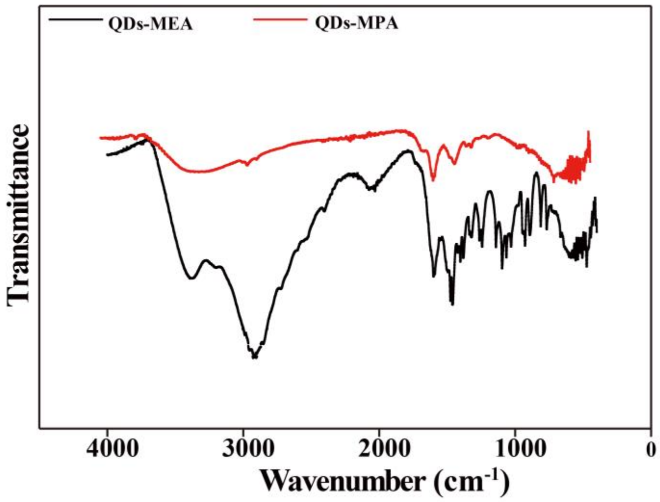

Figure S1. Infrared spectra of the QDs**.**

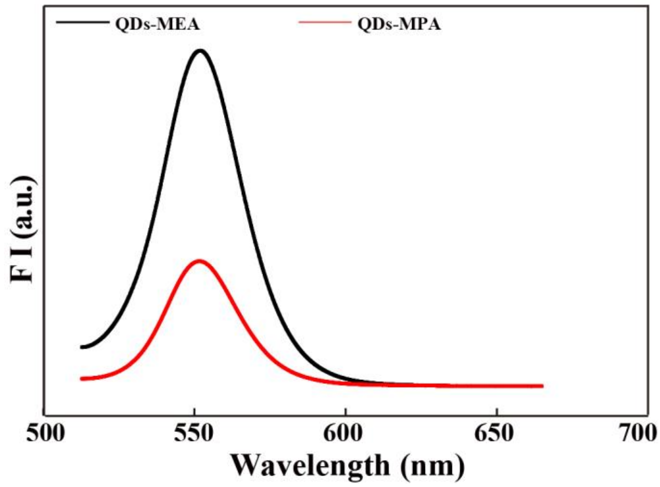

Figure S2. Fluorescence spectra (excitation at 488 nm) of the QDs (25 μg/mL) in water.

 https://doi.org/10.3390/nano15151189

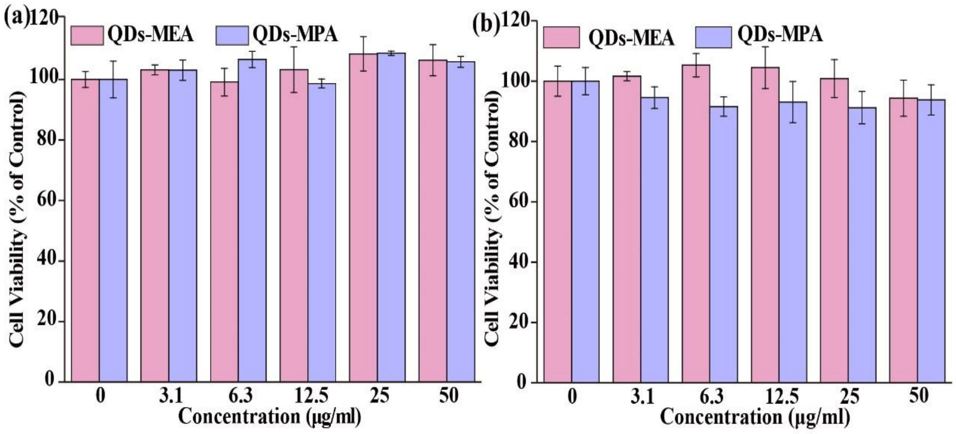

Figure S3. Viability of Raw264.7 cells after exposure to the QDs in culture medium with serum for 24 h (a) and in culture medium without serum for 12 h (b).

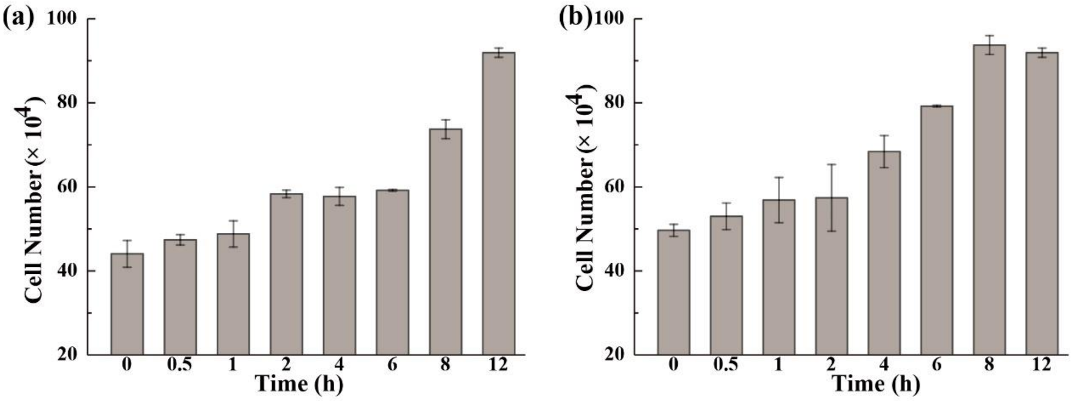

Figure S4. Cell number of Raw264.7 cells after exposed to 25 μg/mL QDs-MEA/QDs-MPA for different times. (a) In culture medium with serum. (b) In culture medium without serum (n=3).

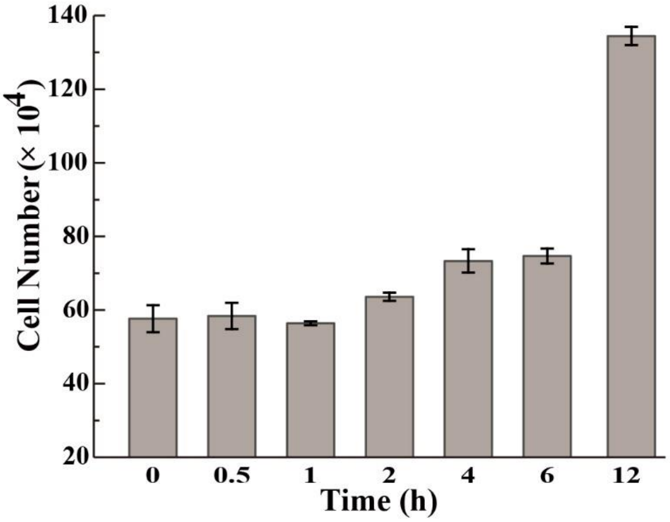

Figure S5. Cell number of Raw264.7 cells during the exocytosis phase. Cells had been preincubated with 25 μg/mL QDs in the medium with serum for 12 h (n=3).

Disclaimer/Publisher's Note: The statements, opinions and data contained in all publications are solely those of the individual author(s) and contributor(s) and not of MDPI and/or the editor(s). MDPI and/or the editor(s) disclaim responsibility for any injury to people or property resulting from any ideas, methods, instructions or products referred to in the content.
